# CRISPR/Cas9 mediated gene-editing of *GmHdz4* transcription factor enhances drought tolerance in soybean (*Glycine max* [L.] Merr.)

**DOI:** 10.3389/fpls.2022.988505

**Published:** 2022-08-19

**Authors:** Xuanbo Zhong, Wei Hong, Yue Shu, Jianfei Li, Lulu Liu, Xiaoyang Chen, Faisal Islam, Weijun Zhou, Guixiang Tang

**Affiliations:** ^1^Zhejiang Provincial Key Laboratory of Crop Germplasm, Institute of Crop Science, Zhejiang University, Hangzhou, Zhejiang, China; ^2^Hainan Institute of Zhejiang University, Sanya, Hainan, China; ^3^Seed Management Station of Zhejiang Province, Hangzhou, Zhejiang, China

**Keywords:** *Glycine max*, HD-ZIP, *GmHdz4*, drought stress, root system architecture

## Abstract

The HD-Zip transcription factors play a crucial role in plant development, secondary metabolism, and abiotic stress responses, but little is known about HD-Zip I genes in soybean. Here, a homeodomain-leucine zipper gene designated *GmHdz4* was isolated. Chimeric soybean plants, *GmHdz4* overexpressing (*GmHdz4-oe*), and gene-editing *via* CRISPR/Cas9 (*gmhdz4*) in hairy roots, were generated to examine the *GmHdz4* gene response to polyethylene glycol (PEG)-simulated drought stress. Bioinformatic analysis showed *GmHdz4* belonged to clade δ, and was closely related to other drought tolerance-related HD-Zip I family genes such as *AtHB12*, *Oshox12*, and *Gshdz4*. The *GmHdz4* was located in the plant nucleus and showed transcriptional activation activity by yeast hybrid assay. Quantitative real-time PCR analysis revealed that *GmHdz4* expression varied in tissues and was induced by PEG-simulated drought stress. The *gmhdz4* showed promoted growth of aboveground parts, and its root system architecture, including the total root length, the root superficial area, and the number of root tips were significantly higher than those of *GmHdz4-oe* even the non-transgenic line (NT) on root tips number. The better maintenance of turgor pressure by osmolyte accumulation, and the higher activity of antioxidant enzymes to scavenge reactive oxygen species, ultimately suppressed the accumulation of hydrogen peroxide (H_2_O_2_), superoxide anion (O^2−^), and malondialdehyde (MDA), conferring higher drought tolerance in *gmhdz4* compared with both *GmHdz4-oe* and NT. Together, our results provide new insights for future research on the mechanisms by which *GmHdz4* gene-editing *via* CRISPR/Cas9 system could promote drought stress and provide a potential target for molecular breeding in soybean.

## Introduction

Soybean (*Glycine max* [L.] Merr.) is an important oilseed crop, used as both food and feed due to its high protein content. China is a traditional soybean-producing country, and the people have long consumed soybeans and processed soybean products. However, a 100 million tons of soybeans need to be imported each year to meet domestic soybean consumption (Sun, 2020), accounting for about 60% of the global soybean trade volume, and external dependence is as high as 80% (Niu et al., 2021). In addition, due to the increase in extreme weather events, the impact of abiotic stresses such as drought, salinity, and high temperature on crop yields and economic losses has increased (Lesk et al., 2016). Soybean is sensitive to water shortage compared with many other crops, requiring about 450–700 mm of water throughout the growth period (Dogan et al., 2007). Especially at podding and filling stages, drought stress can seriously restrict soybean yield, resulting in a loss of about 40% of yield. During the soybean growth period, most areas of China experience different degrees of drought (You et al., 2021). Drought in Northeast China begins in mid-March, and develops into severe and extreme drought conditions from late April to May, which is the growing season for soybean. In addition to water-saving irrigation and raising irrigation efficiency, an effective way to improve soybean yield in water deficit areas is to identify the mechanism of drought tolerance and explore key genes to breed drought-tolerant soybean germplasm.

Homeodomain-leucine zipper (HD-Zip) protein is a class of transcription factor specific to higher plants (Schena and Davis, 1992). Its conserved domain is composed of a homeodomain (HD) and a closely linked leucine zipper (LZ). The HD-Zip proteins can be divided into four subfamilies: I, II, III, and IV. Studies on *Arabidopsis thaliana* (Peterson et al., 2013), rice (*Oryza sativa*) (Zhang et al., 2012), and sesame (*Sesamum indicum*) (Wei et al., 2019) have shown that HD-Zip I is mainly responsible for the regulation of plant growth and development, morphogenesis, and abiotic stress responses (Harris et al., 2011; Gong et al., 2019). For example, genes *AtHB6* and *AtHB7* were strongly induced under drought stress or exogenous abscisic acid (ABA) treatment and stomata were subsequently closed to adapt to these stresses (Perotti et al., 2017). Genes *AtHB7* and *AtHB12* acted as negative feedback regulation factors in the ABA signaling pathway, which influenced lateral root development under water-deficient conditions (Himmelbach et al., 2002; Romani et al., 2016). Expression of *MeHDZ14* was also upregulated under drought and exogenous ABA stress in cassava leaves and roots (Yu et al., 2017). Agalou et al. (2008) found that the expression of *Oshox22* was induced by polyethylene glycol (PEG)-simulated drought but that of *Oshox4*, which was specifically expressed in vascular bundles, was inhibited. In addition, *Oshox4* overexpression led to accelerated gibberellin (GA) metabolism, shortened internode, and prolonged the vegetative growth period in rice. This suggested that *Oshox4* was involved in the negative regulation of GA under drought stress (Zhou et al., 2015b). Overexpression of the *Helianthus annuus* HD-Zip homolog *HaHB4* in wheat increased the number of spikelets, tillers, and fertile florets, and improved grain yield under drought stress (González et al., 2019). Overexpression of *HaHB4* also improved drought tolerance in soybean by increasing xylem area and heat shock protein expression (Ribichich et al., 2020). The mechanisms of drought tolerance were found to be independent of traditional candidate genes such as *RD19* and *DREB1a* (Liu et al., 1998).

There are 36 members of the HD-Zip I transcription factor family in soybean (Chen et al., 2014), among which *GmHB13* was especially induced by water deficit in a drought-tolerant variety (EMBRAPA 48), while expression of *GmHB6* was inhibited in a drought-sensitive variety BR16 (Belamkar et al., 2014). Limited HD-Zip I genes were identified in previous studies. Gene *Gshdz4* (accession number: KHN 42008.1) was reported to participate in drought and salt tolerance regulation (Cao et al., 2017), while *GmHdz4* (Glyma11g06940) was a homologous gene of *Gshdz4* which had a 98.0% amino acid sequence similarity. Therefore, we speculate that *GmHdz4* may also be involved in drought stress response in soybean. The aims of this study were to (1) clone the coding sequence (CDS) of *GmHdz4*, analyze its phylogenetic relationship and the spatio-temporal expression pattern in response to drought stress, (2) analyze the subcellular localization and transcription activity of *GmHdz4*, (3) construct overexpression and CRISPR/cas9 vectors of *GmHdz4* to generate overexpression and knockout chimeric lines to study the function of *GmHdz4*, and (4) reveal the physiological and biochemical mechanisms of drought stress in overexpressing and gene-edited plants. These results may lay a theoretical foundation for further drought-tolerant soybean germplasm innovation.

## Materials and methods

### Plant materials and growth conditions

Soybean (*Glycine max* [L.] Merr.) cv. Tianlong No. 1 was used as the transgenic receptor in this experiment. The wild type and transgenic chimeric plants were grown in a temperature/light-controlled greenhouse at Zhejiang University, Zijingang Campus. All plants were grown under short-day conditions (light/dark of 10/14 h at 26/22°C). The roots, stems, unifoliolate leaves, flowers, and tender pods were taken at V1, R1, and R3 stages, for analysis of tissue-specific expression of *GmHdz4*. The PEG-simulated drought stress was imposed as follows: soybean seeds were germinated in a moist sand bed for 3 weeks. Uniform seedlings were selected and grown using half-strength Hoagland (Xu et al., 2017) solution with 15% PEG6000 (osmotic potential, Ψ_π_ = −0.32 MPa, pH 5.8) to simulate drought stress. The drought-stressed roots were sampled at 0, 3, 6, and 12 h; 1, 2, and 4 days to measure the relative expression of *GmHdz4*. Three biological replicates were used for each analysis. All samples were immediately frozen in liquid nitrogen and stored at −80°C for further analysis.

### Sequence alignment and phylogenetic analysis

The sequences of HD-Zip I family members from *Arabidopsis*, rice, maize, tomato, sunflower, wild soybean, and soybean were screened using BLAST in Phytozome.^1^ Multiple amino acid sequences were aligned using ClustalX 2.0 (Thompson et al., 1994) with default parameters. The phylogenetic tree was produced using MEGA 5.0 software (Tamura et al., 2011) with the neighbor-joining (NJ) method, and the bootstrap value was 1,000 times for each node to test branch reliability.

### Total RNA extraction and cDNA synthesis

The CDS and genome sequence of *GmHdz4* (Glyma11g06940) were obtained and aligned from NCBI,^2^ TAIR,^3^ and Phytozome databases. Total RNA was extracted according to the modified Trizol method (Bekesiova et al., 1999), and RNA integrity was verified by 2% agarose gel electrophoresis. First-strand cDNA was synthesized using a reverse transcription kit (Vazyme, Nanjing, China). Three independent biological replicates were used for RNA extraction and subsequent cDNA synthesis. The CDS of *GmHdz4* was amplified by gene-specific primers GmHdz4_CDS_F (5′-ATGAATCATCGACCACCTTTCC-3′) and GmHdz4_CDS_R (5′-CAGATTAATCCATTCCATGCCG-3′) using Taq Master Mix (Vazyme, Nanjing, China). The target PCR products were cut and recycled with a gel extraction kit (Sangon Biotech, Shanghai, China) after separation by 2% agarose gel electrophoresis. The product was cloned into pUCI-T vector (Sangon Biotech, Shanghai, China) then transformed to *Escherichia coli* DH5α competent cells by the freeze–thaw method (Knopf, 1976). The selected single colonies were sequenced and stored at −20°C for subsequent experiments.

### Real-time fluorescent quantitative PCR (qRT-PCR) analysis

The qRT-PCR was performed using ChamQ SYBR qPCR Master Mix (Vazyme, Nanjing, China), according to the manufacturer’s instruction. Specific primers designed for *GmHdz4* were GmHdz4Q_F (5′-GACCACCTTTCCAAGACCACA-3′) and GmHdz4Q_R (5′-AGCTCCATTGCCAGCCTATC-3′). The normalizing reference gene was *Actine11* (Xue et al., 2012). Abnormal CT values (> 0.2 + CT_median_) were eliminated, and relative expression levels were calculated according to the 2^−ΔΔCT^ method (Livak and Schmittgen, 2001). All samples were selected randomly under the same greenhouse conditions. Three technical replicates for each biological replicate were used in qRT-PCR analysis.

### Plasmid construction

For overexpression plant transformation, the full-length CDS of *GmHdz4* isolated from soybean root tissue was amplified by PCR using premiers: GmHdz4-plus-F (5′-CAGTGAATTCCTGGACGTCCGTACGTTCGA-3′) and GmHdz4-plus-R (5′-CGATGAATTCCGGCGCAAAAATCACCAGTC-3′). The amplification products were inserted into the pTF102 vector *EcoRI* site to generate pTF102–*GmHdz4* vector, containing a *bar* gene under the control of the CaMV35S promoter for the herbicide-based plant selection (Supplementary Figure S2A). For gene-editing plant transformation, the target sequence of *GmHdz4* (Supplementary Figure S2C) was predicted by CRISPR-P^4^ (Lei et al., 2014) and the sgRNA sequence was 5′-GTCCGAAAGAAAGGATAGGC-3′. Specific designed oligo probes were 5′-GGGTTGGTCCGAAAGAAAGGATAGGC-3′ and 5′-AAACGTCCGAAAGAAAGGATAGGCCA-3′. The amplified dimer was inserted into the pBGK041 vector according to the manufacturer’s instructions to generate gene-editing pBGK041–*GmHdz4* vector. The pBGK041 vector (Biogle, Jiangsu) contained Cas9 cassettes, driven by S35, and the inserted sgRNA was driven by GmU6 promoters (Supplementary Figure S2D). The plasmids of pTF102–*GmHdz4* recombinant vector and pBGK041–*GmHdz4* gene-editing vector were transformed into *Agrobacterium rhizogenes* K599 by the freeze–thaw method (Knopf, 1976), respectively.

### Subcellular localization and transcriptional activation analysis

For subcellular localization, the recombinant vector pCAMBIA1302–*GmHdz4* expressing the *GmHdz4*–GFP fusion protein and the positive marker vector pBWD-NLSmKAT expressing a nuclear-localized red fluorescent protein (Zoonbio, Nanjing, China) were transferred into *Agrobacterium tumefaciens* GV3101 by electroporation, respectively. After incubating at 30°C for 12 h, single colonies were picked out and cultured at 170 rpm/min for 1 h. A needle-free syringe was used to inject the resuspended bacterial solution under the epidermis of *Nicotiana tabacum* leaves, and this was marked. The *GmHdz4*–GFP fusion protein was examined 2 days after culture under weak light using a confocal laser scanning microscope (FV1000; Olympus, Japan). This experiment was repeated three times independently.

For transcriptional activation analysis, the experiment was based on the GAL4 yeast two-hybrid (Y2H) system (Chien et al., 1991), and the *GmHdz4* gene was introduced into pGBKT7 vector. Plasmids pGBKT7-Lam and pGADT7-T were co-transformed into Y2HGold yeast as a negative control, and plasmids pGBKT7-53 and pGADT7-T were used as a positive control. Transcription activation activity was analyzed following the user manual of Takara Bio USA, Inc.

### Hairy roots system generation and identification

Healthy and vigorous seeds of Tianlong No. 1 were selected, sterilized by chlorine gas, and germinated in sterile vermiculite for 5 days. After the hypocotyl grew about 2–3 cm length, 200 μl resuspended *A*. *rhizogenes* solution (OD600 ≈ 0.6) carrying recombinant plasmid was injected into each hypocotyl about 2 mm below the cotyledon node according to Kereszt et al. (2007). The *A*. *rhizogenes* without the target gene was used as a control. The photoperiod was controlled for 6 h of light and18 h of dark, temperature was maintained at 28°C, and relative humidity was about 70%. The hypocotyl and primary roots were excised when enough hairy roots were induced 1 week after injection. The chimeric seedlings were transplanted into half-strength Hoagland nutrient solution. The chimeric seedlings containing pTF102–*GmHdz4* and pBGK041–*GmHdz4* vectors were labeled *GmHdz4-oe* and *gmhdz4*, respectively, and seedlings without the target gene plasmids were labeled non-transgenic (NT).

The hairy roots of *GmHdz4-oe* chimeric seedlings were verified by PCR amplification using specific primers GmHdz4_CDS, which were mentioned above. The hairy roots of *gmhdz4* chimeric seedlings were verified by PCR using *Cas9* gene specific primers (5′-ACAAGGTGAGCGTTGTTTAT-3′ and 5′- CATGATGGTGGTATGTTTCG-3′). And the target site on the *GmHdz4* CDS of each *gmhdz4* hairy root was sequenced. The hairy roots from the independent chimera seedling line were collected and mixed to detect the expression of target sequence on *GmHdz4* CDS. Three independent transgenic lines as biological replicates were performed.

### Identification of *GmHdz4* function by PEG-simulated drought stress

The positive overexpressed and gene-editing chimeric soybean seedlings were transplanted into half-strength Hoagland solution containing 15% PEG6000 (pH 5.8) for drought stress treatment. The phenotypic traits and physiological indexes were determined at 0, 6, and 12 h; 1, 2, 4, and 8 days after PEG treatment. Fresh root samples were immediately frozen in liquid nitrogen and stored at −80°C for further experiments. The following experiments had three biological replicates at least.

### Biomass determination

After 8 days of PEG treatment, the fresh shoots and roots were dried for 3 h at 105°C and then for another 24 h at 80 ± 1.5°C to obtain a constant dry weight for the measurement for dry biomass. The ratios of root mass to shoot mass were then calculated.

### Root system architecture analysis

The chimeric seedlings were cleaned in nutrient solution 8 days after PEG treatment. The hairy roots were scanned by Epson Perfection V850 Pro. The root system architecture was analyzed using WinRHIZO software (Win et al., 2016).

### Hairy root activity determination

The root activity of each sample was measured by improved 2,3,5-triphenyl tetrazolium chloride (TTC) method (Higa et al., 2010). A microplate reader was used to detect the OD value of formazan at a wavelength of 485 nm. The TTC-reducing activity was converted to represent the root activity.

### Osmoprotectant estimation

The content of free proline was determined according to Bates et al. (1973) and of soluble sugar according to Bailey (1958). The absorbance was measured using a microplate reader (Synergy™, BioTek).

### Determination of hydrogen peroxide (H_2_O_2_), superoxide anion radical (O^2−^), and malondialdehyde (MDA) contents and antioxidant enzyme activities

The H_2_O_2_ and O^2−^ contents in hairy roots were measured according to Alexieva et al. (2001) using reactive oxygen species (ROS) content reagent kits (Solarbio, Beijing). The enzyme activities of superoxide dismutase (SOD, EC1.15.1.1), peroxidase (POD, EC 1.11.1.7), and catalase (CAT, EC 1.11.1.6) were measured by NBT illumination, guaiacol reaction, and oxidation–reduction methods, respectively (Wang et al., 2021). The MDA was measured according to Heath and Packer (1968) and Tang et al. (2013).

### Statistical analysis

All data are presented as the mean values with the standard deviation of three replications in figures. Statistical analysis was performed using EXCEL 2019 (Microsoft Inc., Redmond, WA, United States) and SPSS 20 (SPSS Inc., Chicago, IL, United States). Significant differences were determined using one-way ANOVA and Duncan’s multiple range tests. The differences between treatments were considered significant at *p* < 0.05. Prism7 (GraphPad Inc., San Diego, CA, United States) was used to visualize the data.

## Results

### *GmHdz4* cloning and phylogenetic analysis

**Figure 1:**
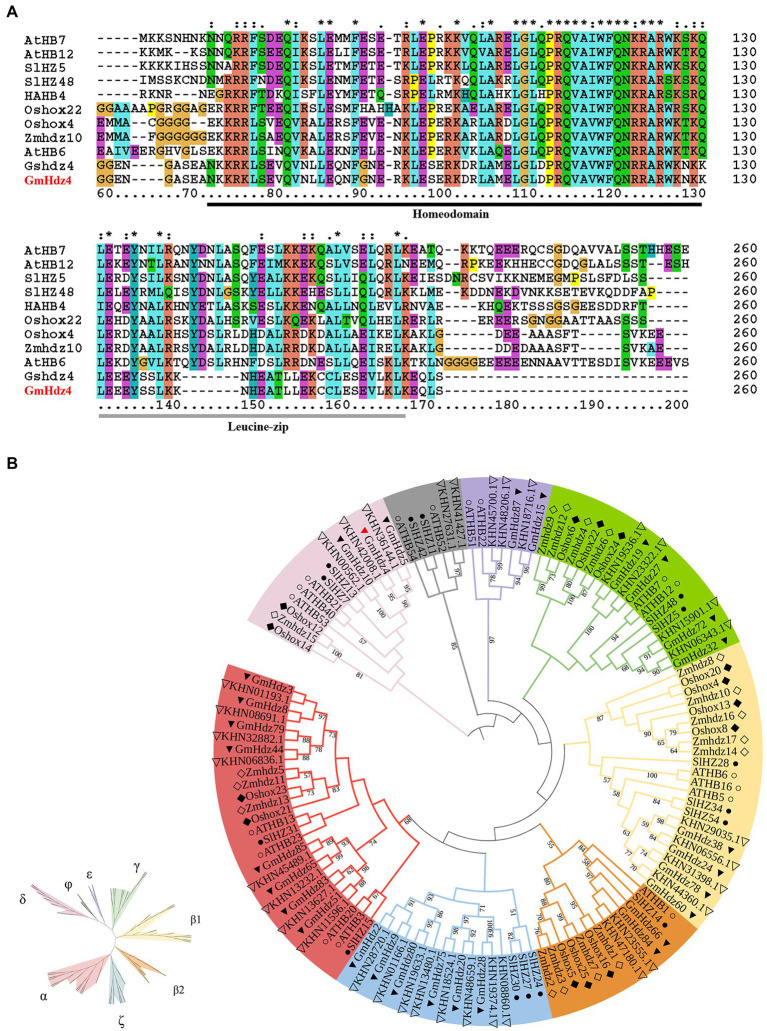
Sequence alignment and phylogenetic analysis. **(A)** Multiple sequence alignment for GmHdz4 and other drought stress-related HD-Zip I proteins. HD and LZ domains are underlined by black and gray, respectively. The corresponding IDs of Oshox22, Oshox4, AtHB7, AtHB12, AtHB6, Gshdz4, GmHdz4, HAHB4, Zmhdz10, SlHZ48, and SlHZ5, are Q7XUJ5.2, Q6K498.1, P46897.2, Q9M276.1, P46668.1, AOG74801, Glyma11g06940, XP_022022563, AFT92045, XP_004245456, and XP_004230017, respectively. **(B)** Phylogenetic analysis of HD-Zip I transcription factors from *Arabidopsis*, rice, maize, tomato, soybean, and wild soybean. The phylogenetic tree was built using NJ method in MEGA. Bootstrap support is indicated on the branches. Genes from each of the species are marked with different symbols: *Arabidopsis* (○), rice (■), maize (□), soybean (▼), wild soybean (▽), and tomato (●).

The CDS sequence of *GmHdz4* (Glyma11g06940) was amplified by high-fidelity PCR using cDNA from total RNA of soybean cultivar Tianlong No. 1 as a template. The PCR product was cloned into pUCI-T vector to obtain pUCI–*GmHd4* (Supplementary Figure S1) and sequenced. The sequencing result was completely consistent with the NCBI database. Gene *GmHdz4* contained a 648-bp open reading frame encoding a protein of 215 amino acids; the molecular weight of GmHdz4 protein was 25.11 kDa, and the isoelectric point was 6.48. Sequence analyses revealed that most of the GmHdz4 amino acid sequence formed hydrophilic regions except for the aa10–24, aa64–72, and aa88–102, and nine out of 12 sites that may undergo phosphorylation modification were located in these regions. The results of multiple sequence alignment showed that GmHdz4 had a highly conserved HD compared with other HD-Zip I transcription factors, which was related with drought stress in *Arabidopsis*, rice, maize, tomato, wild soybean, and sunflower. The LZ domain was also relatively conserved, with only individual leucine sites mutated to threonine or valine (Figure 1A). The 3D model of GmHdz4 protein predicted by SWISS MODEL Workspace showed that the HD domain folded into three α-helices. This spatial conformation provided the conditions to recognize and bind the promoter elements of the target genes, and is consistent with the typical structural features of HD-Zip I transcription factors.

Phylogenetic analysis was performed among HD-Zip I subfamily members in *Arabidopsis*, rice, maize, tomato, wild soybean, and soybean. The clades in the tree were labeled according to previous studies in *Arabidopsis* and rice. The HD-Zip I subfamily members were clustered into eight subclasses: α, β1, ζ, γ, ɛ, β2, δ, and φ (Figure 1B). The distribution showed an obvious species preference. Six species contained HD-Zip I protein clades α, β1, β2, γ, and δ while only dicotyledons such as *Arabidopsis*, tomato, and soybean included clades ζ, ɛ, and φ. The results suggested that the main characteristics of this HD-Zip I subfamily were generated before the dicot–monocot split and differentiated continually after the split. Gene *GmHdz4* was located in clade δ, and was closely related to drought stress-related HD-Zip I genes such as *AtHB12*, *Oshox12*, and *Gshdz4*.

### *GmHdz4* spatio-temporal and tissue-specific expression

**Figure 2:**
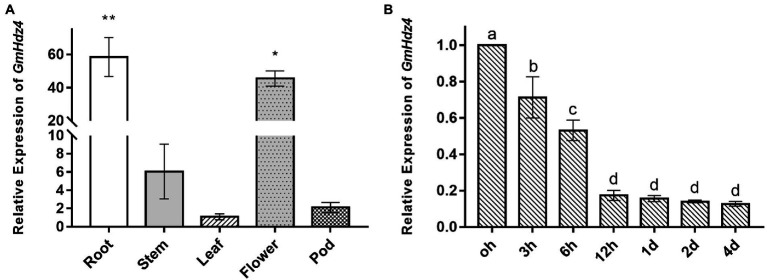
Tissue-specific and spatio-temporal expression of *GmHdz4*. Expression pattern of *GmHdz4* in various tissues in soybean **(A)** and PEG-induced expression pattern of *GmHdz4* in root **(B)**. Significant differences are indicated by ^*^ and ^**^ for *p* < 0.05 and *p* < 0.01, respectively, according to Duncan’s test in panel **A**; values with different letters (a–d) significantly differ at *p* < 0.05 according to Duncan’s test in panel **B**.

The relative expressions of *GmHdz4* in roots, stems, leaves, flowers, and pods significantly differed with the main expression in flowers and roots (Figure 2A). Expression of *GmHdz4* in roots was about 60-fold higher than that in leaves, and 1.3-fold higher than that in flowers. The relative expression of *GmHdz4* in roots showed a stepwise significant decrease within 12 h after 15% PEG6000 (Ψ_π_ = −0.32 MPa) simulated drought stress (Figure 2B). The relative expression of *GmHdz4* was reduced by 47.0% at 6 h after drought stress, and dropped to about 18% by 12 h after drought stress. Later, *GmHdz4* expression decreased slightly and basically remained at 16% before treatment. The results suggested that *GmHdz4* expression was inhibited by drought in soybean roots.

### *GmHdz4* subcellular localization and transcriptional activity

**Figure 3:**
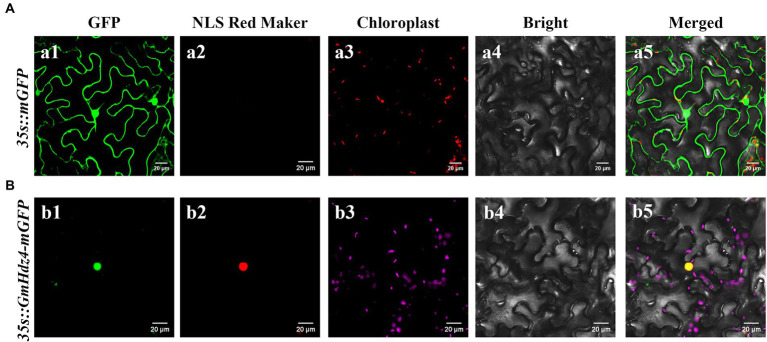
Subcellular localization analysis of *GmHdz4*. The fusion plasmid (35S::GmHdz4–GFP), the negative control plasmid (35S::mGFP), and pBWD-NLSmKAT expressing an NLS Red Marker protein were transiently transformed into tobacco epidermal cells. Images were taken in the dark field for green fluorescence (a1, b1), red fluorescence (a2, b2), and chloroplast fluorescence (a3, b3), while the outline of cells (a4, b4) and merged image (a5, b5) were photographed in a bright field. Microscopy detections of 35S::mGFP were displayed in penal **(A)**, and the detections of 35S::GmHdz4–mGFP were displayed in penal **(B)**. Bars represent 20 μm.

Gene *GmHdz4* might target the nucleus according to its amino acid sequence, which contained an NLS sequence at the region of aa35–60. The fluorescence signal of *GmHdz4* was only detected in the nucleus when tobacco cells were injected with a 35 s::*GmHdz4*–m*GFP* fusion construct and the NLS Red Marker (Figure 3B). However, the GFP signal was found throughout the cell using 35 s::mGFP as a control (Figure 3A).

**Figure 4:**
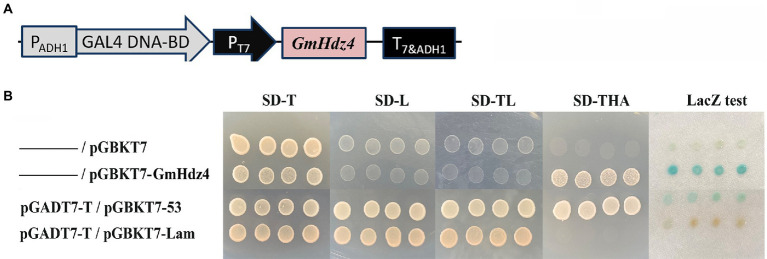
Transcription activity analysis of *GmHdz4*. **(A)** The construct of pGBKT7–GmHdz4; **(B)** ADE2 and HIS3 reporter assay, and galactosidase (LacZ) assay. pGADT7-T/pGBKT7-53 and pGADT7-T/pGBKT7-Lam vector groups were used as positive and negative controls, respectively.

The pGBKT7–*Gmhdz4* recombination vector was delivered into yeast strain Y2HGold to identify the transcriptional activation activity of *Gmhdz4* (Figure 4A). The interaction between pGBKT7-Lam and pGADT7-T was the negative control, while the interaction between pGBKT7-p53 and pGADT7-T was the positive control. Yeast cells carrying the pGBKT7 empty plasmid and the negative control could not grow on SD/Trp-/His−/Ade- medium (Figure 4B); however, yeast cells carrying pGBKT7–*GmHdz4* and the positive control grew well. This showed that the fusion expression of GmHdz4 and GAL4 DNA-binding domain could activate reporter genes *ADE2* and *HIS3*. Meanwhile, pGBKT7–*GmHdz4* also showed high galactosidase activity in the *LacZ* assay. The results indicated that *GmHdz4* regulated transcription activities in the nucleus, consistent with two basic characteristics of transcription factors.

### The *gmhdz4* mutant were more tolerant to drought stress

The *A*. *rhizogenes*-mediated transformation system was used to generate *GmHdz4* overexpression and gene-editing chimeric soybean in order to explore *GmHdz4* function. The fragments of *GmHdz4* CDS with correct size could be amplified from most hairy roots of the three random selected *GmHdz4*-oe independent lines (Supplementary Figure S2B), that meant most individual hairy roots of *GmHdz4*-oe lines were successfully transformed. Similarly, majority hairy roots of *gmhdz4* lines have *Cas9* gene sequence (Supplementary Figure S2E), and there were three mutation types including a single base deletion on the 256 bp site, a single base insertion between the 256 bp site and the 257 bp site, and a four bases deletion of 254 bp to 257 bp (Supplementary Figure S2F). However, the target site of some transformed hairy roots were not edited successfully. In addition, the relative expression of *GmHdz4* in *GmHdz4*-oe hairy roots was 5.1–8.8-fold higher than that for NT, whereas *GmHdz4* expression in *gmhdz4* hairy roots was 2.7–5.3-fold lower than that of NT (Supplementary Figure S2G).

**Figure 5:**
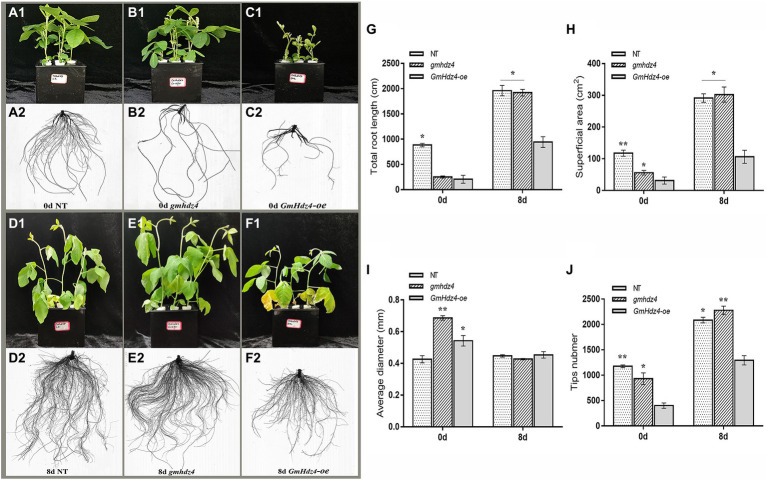
The phenotype and root system architecture among NT, *gmhdz4* chimeric lines, and *GmHdz4-oe* chimeric lines. Phenotypic comparison of transgenic hairy roots chimera and NT soybeans before and after PEG treatment **(A1–F2)**. The root system architecture analysis of total root length **(G)**, superficial area **(H)**, average diameter **(I)**, and tip numbers **(J)**. NT, non-transgenic soybeans; *gmhdz4*, gene-editing chimeric line; *GmHdz4-oe,* overexpression *GmHdz4* chimeric line. At least three biological replicates were performed, and the data before (0d) and after (8 days) treatment were statistically analyzed, respectively. Significant differences are indicated by ^*^ and ^**^ for *p* < 0.05 and *p* < 0.01, respectively, according to Duncan’s test.

**Figure 6:**
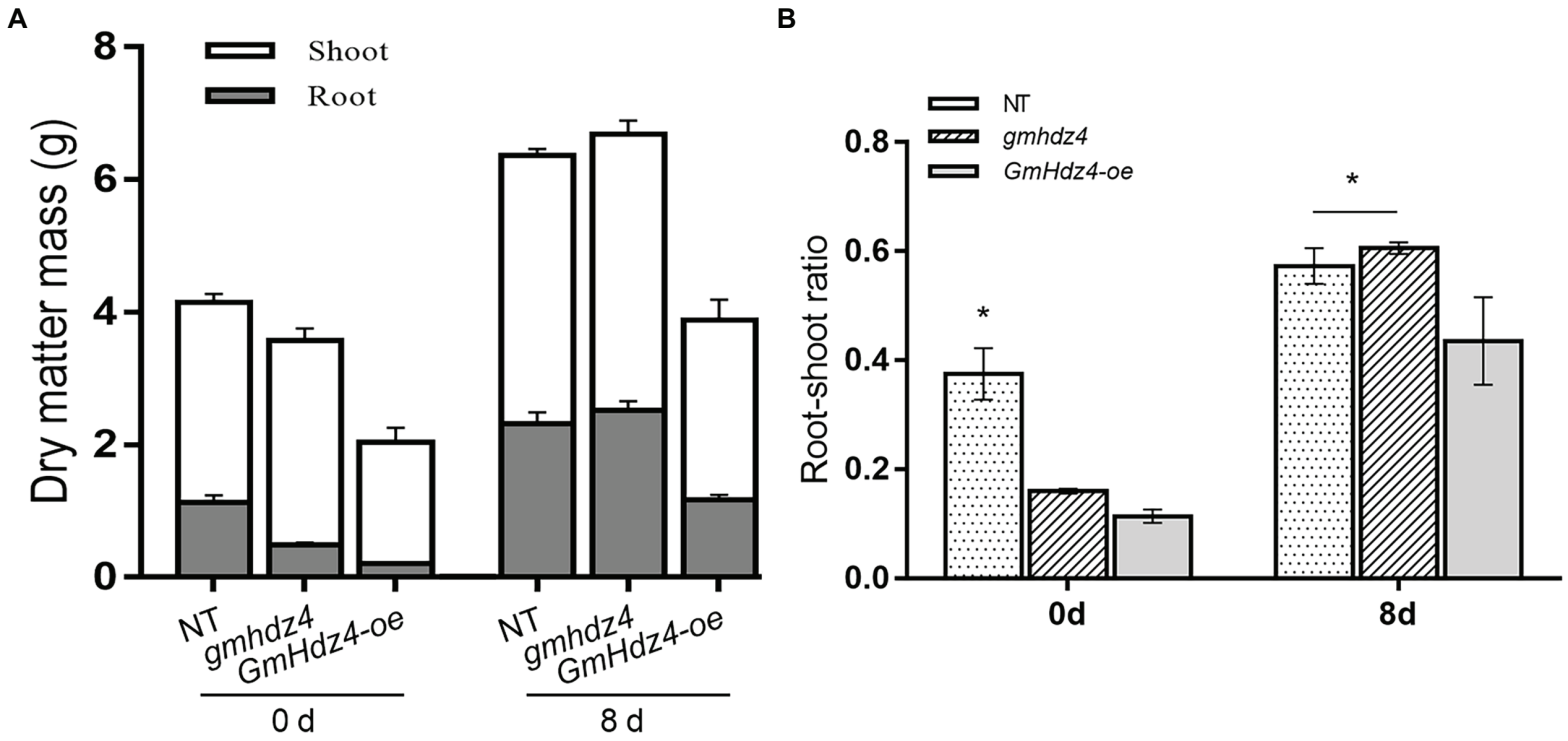
The dry matter mass **(A)** and root–shoot ratio **(B)** of each line before and after PEG treatment. Significant differences are indicated by ^*^ for *p* < 0.05, according to Duncan’s test.

The phenotypes obviously differed among NT, *GmHdz4-oe*, and *gmhdz4* at 8 days after 15% PEG6000 simulated drought stress. The leaves were green and vigorous in NT (Figure 5A1) and *gmhdz4* (Figure 5B1) before PEG treatment, and hairy roots developed normally (Figures 5A2,B2). However, leaves in *GmHdz4-oe* (Figures 5C1,C2) were etiolated and short, and hairy roots were stunted. Eight days after PEG treatment, most leaves in *GmHdz4-oe* were wilted and yellow, and the first trifoliolate leaf was dried and shed (Figures 5F1,F2). The dry weight of aboveground parts showed no significant difference between *gmhdz4* and NT after PEG treatment, while that of *GmHdz4-oe* was relatively low (Figure 6A). Eight days after PEG treatment, *gmhdz4* had the greatest root dry weight among the three chimeric lines with an average of 2.52 g/plant, which increased 411.2% before treatment. Emphatically, root dry weight in *GmHdz4-oe* was clearly the lowest, with less than one-half of that of *gmhdz4* and NT. Additionally, the root–shoot ratio in descending order was *gmhdz4*, NT, and *GmHdz4-oe* (Figure 6B).

The hairy root system in *GmHdz4-oe* was much weaker than that of NT (Figures 5D1,D2), while the root system in *gmhdz4* developed well (Figures 5E1,E2). Analysis of root system architecture showed that the total root length (Figure 5G), superficial area (Figure 5H), and tip number (Figure 5J) in *gmhdz4* were significantly higher than that of *GmHdz4-oe,* but not average diameter (Figure 5I) after PEG treatment. The total root length in *gmhdz4* increased from 240 cm (before PEG treatment) to 2,100 cm (after PEG treatment), an increase of 775%, which was 5.8 times that of NT. The root surface area in *gmhdz4* also increased from 55.97 to 302.33 cm^2^ and the number of root tips in *gmhdz4* increased from about 900 to 2,300, with a significantly higher increase than that of NT and *GmHdz4*-oe. These results suggested that gene editing of *GmHdz4* promoted the root morphogenesis under PEG treatment and enhanced drought tolerance in soybeans.

### Accumulation of osmoprotectants, and hairy root activity enhanced in *gmhdz4* mutant

**Figure 7:**
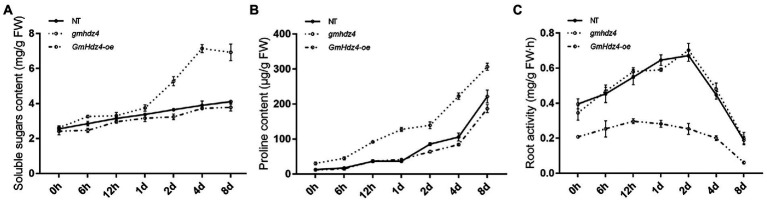
Soluble sugar content **(A)**, proline content **(B)**, and root activity **(C)** after PEG treatment. The straight line, dotted line, and dash-dotted line represent non-transgenic soybeans (NT), *gmhdz4* chimeric line, and *GmHdz4-oe* line, respectively.

There was no significant difference in soluble sugar and free-proline contents among hairy roots of *GmHdz4-oe*, *gmhdz4*, and NT lines before PEG treatment (Figure 7). With the prolongation of PEG-simulated drought stress, the soluble sugar and free-proline contents increased gradually among the three different lines (Figures 7A,B). However, the osmoprotectants in *gmhdz4* increased significantly more than in *GmHdz4-oe* and NT. Eight days after PEG treatment, the soluble sugar content in *gmhdz4* was 1.76 and 1.84 times higher than that of NT and *GmHdz4-oe*, respectively (Figure 7A); correspondingly, the proline content was 1.48 and 1.63 times higher (Figure 7B). This suggested that *gmhdz4* obtained stronger osmotic accumulation ability under drought stress. Additionally, the root activity of *gmhdz4* and NT showed no significant difference, while that of *GmHdz4-oe* significantly decreased after PEG treatment. The results suggested that gene editing of *GmHdz4* maintained a high osmotic potential in hairy roots to ensure lower cellular water potential under drought stress, so that roots of *gmhdz4* maintained normal physiological activity to cope with drought stress.

### The *gmhdz4* mutant promotes ROS scavenging capacity under drought stress

**Figure 8:**
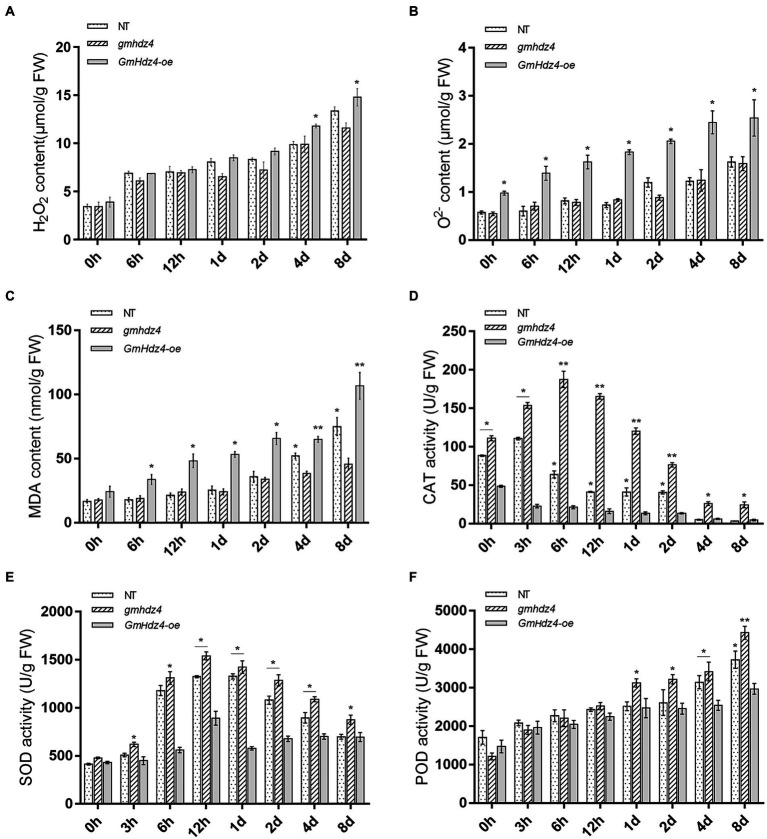
Knockout of *GmHdz4* promotes ROS scavenging in response to drought stress. Contents of H_2_O_2_ **(A)**, O^2−^ **(B)**, and malondialdehyde (MDA) **(C)**, and activities of catalase (CAT) **(D)**, superoxide dismutase (SOD) **(E)**, and peroxidase (POD) **(F)** were tested to assess the antioxidation systems under drought stress. Significant differences are indicated by ^*^ and ^**^ for *p* < 0.05 and *p* < 0.01, respectively, according to Duncan’s test.

Accumulation of ROS increased gradually with continuing PEG treatment in soybean hairy roots. There was no significant difference in H_2_O_2_ contents in the hairy roots of NT and *gmhdz4* in the first 3 days after PEG treatment. However, the O^2−^ content in *GmHdz4-oe* was significantly higher than that of NT and *gmhdz4*. Subsequently, H_2_O_2_ accumulated rapidly in *GmHdz4-oe* chimeric lines (Figure 8A). The differences in O^2−^ contents between *GmHdz4-oe* and the other two lines reached a maximum of about 2.1 times at 4 days after PEG treatment (Figure 8B). Eight days after PEG treatment, H_2_O_2_ content in *gmhdz4* was only 12.0 μmol/g and was the lowest, compared with *GmHdz4-oe* and NT. Correspondingly, the MDA content was also significantly lower in *gmhdz4* chimeric lines (Figure 8C).

The antioxidase activities of hairy roots after PEG treatment are shown in Figures 8D–F. The CAT and SOD enzyme activities initially increased and then declined within 8 days after PEG treatment (Figures 8D,E). The CAT activity in *gmhdz4* and NT reached a maximum at 6 and 3 h after PEG treatment, respectively. The CAT activity in *gmhdz4* was higher than that of NT, while *GmHdz4-oe* maintained the lowest CAT activity during the PEG treatment (Figure 8D). The SOD activity in NT and *gmhdz4* was significantly higher than that of *GmHdz4-oe* from 6 h to 4 days after PEG treatment, with a peak at 12 h (Figure 8E). In addition, POD activity increased with extension of PEG treatment. The POD activity in *gmhdz4* was significantly higher than that of NT and *GmHdz4-oe* from 1 days after PEG treatment, and POD activity in NT was also higher than that of *GmHdz4-oe* during 4–8 days after PEG treatment (Figure 8F). The results suggested that *gmhdz4* created by CRISPR/Cas9 could stimulate the activities of antioxidases in soybean hairy roots under drought stress, and was beneficial to maintain the redox balance of the membrane system.

## Discussion

The regulation of abiotic stress response by transcription factors is a hot topic in plant research. Most of the reported transcription factors associated with drought resistance in soybean are concentrated in the MYB, WRKY, bZIP, bHLH, and NAC families, accounting for about 63% of the total (Chai et al., 2015; Khan et al., 2018). In recent years, studies on *Arabidopsis*, rice, maize, and tomato showed that a plant-specific transcription factor, HD-Zip, was also involved in the response to abiotic stress. It was documented that 75% of HD-Zip genes in sesame were associated with drought and salt stress (Wei et al., 2019), but few studies in soybean were reported. In the present study, a novel HD-Zip I subfamily gene, *GmHdz4*, was cloned from the cDNA library of soybean cultivar Tianlong No. 1. It encodes a typical HD-Zip I protein which contains a conserved HD and a closely linked LZ domain (Ariel et al., 2007). The subcellular localization (Figure 3) and yeast hybridization assay (Figure 4) showed that *GmHdz4* had the function of nuclear localization and transcription activation. Phylogenetic analysis (Figure 1B) showed that *GmHdz4* was clustered in clade δ with other drought-tolerant HD-Zip I genes such as *AtHB7*, *SlHZ48*, and *SlHZ5* (Jiang, 2017). Compared with rice, maize, and other gramineous crops, soybean HD-Zip I members were more numerous and most of them had paralogous genes. Our study confirmed that soybean underwent two duplication events and genome expansion in a species-specific manner after mono–dicot differentiation (Kong et al., 2007; Jin et al., 2017). This approach is one of the main evolutionary mechanisms for generating new genes that help plants adapt to environmental stresses such as drought (Skirycz and Inze, 2010).

We found that *GmHdz4* was expressed in various organs in soybean, and notably its expression level was significantly higher in roots and flowers (Figure 2A). Whereas, Belamkar et al. (2014) found that *GmHdz4* homologs, *GmHdz5* and *GmHdz10*, were only expressed in flowers. Transcriptome studies showed that *GmHdz38* was highly expressed in the whole plant; *GmHdz24*, *GmHdz60*, and *GmHdz84* were mainly expressed in roots and flowers; and *GmHdz66* was only expressed in roots (Libault et al., 2010a, 2010b; Belamkar et al., 2014). The complexity role of expression in the HD-Zip I family determines their diversity of gene functions. Expressions of *GmHdz19* and *GmHdz27* increased under drought stress, while those of *GmHdz24*, *GmHdz84*, and *GmHdz66* were significantly downregulated (Chen et al., 2014). Overexpression of *Zmhdz10* improved drought and salt tolerance in transgenic maize by regulating the ABA signaling pathway (Zhao et al., 2014). Furthermore, expression levels of *ATHB7* and *ATHB12* in *Arabidopsis* seedlings were upregulated 12–25 times after hydropenia or exogenous ABA treatment (Söderman et al., 1996; Lee et al., 2001; Valdes et al., 2012). Expressions of *ATHB40*, *ATHB21*, and *ATHB53*, which are orthologs of *GmHdz4*, were also upregulated by at least twice (Henriksson et al., 2005). We found that *GmHdz4* expression decreased by about 82% at 12 h after PEG-simulated drought stress (Figure 2B), with a similar response pattern to root-specifically expressed gene *GmHdz66* (Chen et al., 2014). The root system is the most important organ of plants in response to drought signals. Expression of *GmHdz4* was suppressed in roots by drought, suggesting that this transcription factor gene may function in soybean roots in response to drought stress.

The plant root system architecture is plastic and dynamic. Plants make their root systems stronger and more developed in order to absorb water. The more lateral roots formed and the wider they are distributed, the more drought resistant is the plant (Battisti and Sentelhas, 2017). In general, the root system is the first organ to sense changes in soil water content, when plants suffer water deficit and produce a large number of root signals for transport to the aboveground parts. Thus, plants can promote root growth and development by regulating the distribution of assimilated substance to avoid hydropenia in the early stage of drought stress (Gilbert et al., 2011; Hu and Xiong, 2014). However, severe drought can inhibit growth of the root system and affect the formation of final yield (Huang et al., 2014; Ye et al., 2018). In our study, *gmhdz4* chimeric plants generated by CRISPR/Cas9 promoted hairy root growth to some extent, while overexpression of *GmHdz4* chimeric plants inhibited the development of hairy roots under drought stress (Figures 5A1–F2). The root system architecture results suggested that *GmHdz4* negatively regulated the development of lateral roots, since the root surface area and root tip numbers of *gmhdz4* increased significantly (Figures 5G–J).

Among the HD-Zip I subfamily in *Arabidopsis*, only *AtHB7* and *AtHB12* were found to be related to lateral root development under water deficit conditions, and played a negative feedback regulation role in the ABA pathway (Romani et al., 2016). The roots of the *athb7/athb12* double mutant were shorter than the wild type (Ré et al., 2014). The *AtHB53* gene, a *GmHdz4* homolog expressed in the root apical meristem, was induced by hyperthermia and osmotic stress, and played an important role in auxin/cytokinin signaling pathways in roots (Son et al., 2005). A recent study by Perotti et al. (2021) showed that most of the HD-Zip members related to root development belonged to subfamilies III and IV. Gene *AtHDG11* was involved in the auxin signaling pathway, which regulated drought resistance, and root biomass increased significantly in overexpressing plants. Expression of *IAA28* in mutant *hdg11* was downregulated and exhibited a drought-sensitive root phenotype (Rogg et al., 2001; Nakamura et al., 2006). In addition, *AtHB10* acted downstream of MYBs and was a negative regulator of root hair formation (Wang et al., 2010). Smetana et al. (2019) found downregulated expression of *AtHB8*, *AtHB9*, and *AtHB15* after drought stress, and their functions for differentiation and recognition of xylem were observed in the apical mature zone of roots. Gene *MtHB1* was strongly expressed in root tips and regulated lateral root formation (Ariel et al., 2010). Wild soybean *GsHdz4* was highly similar to *GmHdz4*, and regulated alkaline and osmotic stresses through different pathways. This positively regulated HCO_3_^−^-stress tolerance, but sensitivity to osmotic stress was aggravated (Cao et al., 2017). This is consistent with the root phenotype of the soybean line overexpressing *GmHdz4* in the present study. Overexpression of *GmHdz4* affected aboveground growth and biomass accumulation by suppressing differentiation and elongation of lateral roots (Figure 6), resulting in plants with diminished drought tolerance. However, the molecular basis of *GmHdz4* regulating root morphology needs further study.

In addition to affecting root system architecture, the physiological mechanism of *GmHdz4* reducing the drought tolerance of soybean was further explored. Overexpression of *GmHdz4* resulted in decreased osmotic regulation capacity, accumulation of proline and soluble sugar were blocked (Figures 7A,B), and oxidative damage in hairy roots was aggravated under drought stress (Figures 8A–C). Conversely, gene-editing *GmHdz4* alleviated these damages to some extent. Free proline and soluble sugars are the most common osmoregulation substances in plants (Liu and Zhu, 1997). The content of free proline in plants is very low under normal circumstances, but it accumulates rapidly under abiotic stress such as drought and is positively correlated with plant tolerance to stress (Gilmour et al., 2000; Du et al., 2020). Varieties with stronger osmotic regulation ability had less yield reduction under drought stress (Blum, 2017). The soybean osmotic regulation inhibited by *GmHdz4* was similar to that by *MtHB2*, which reduced proline synthesis and accumulation by inducing expression of a proline dehydrogenase gene *ProDH*, which led to adverse effects on drought tolerance (Song et al., 2012). In addition, proline acts as a molecular chaperone to stabilize protein structure and reduce oxidative damage to cells (Székely et al., 2008). This oxidative damage is a complication of plants suffering from drought stress, which is manifest in significant accumulation of ROS and plasma membrane oxidative damage marker, MDA (Mittler, 2002; Verslues et al., 2007; Sun et al., 2018). We found that the activities of antioxidases (e.g., CAT, SOD, and POD) in *gmhdz4* increased rapidly and rhythmically under drought stress (Figures 8D–F). It is crucial to reduce the content of H_2_O_2_ and O^2−^ and relieve the oxidative senescence of roots. In contrast, these antioxidases in hairy roots overexpressing *GmHdz4* were relatively sluggish in response to drought, and the ROS could not be scavenged promptly, leading to continuous accumulation of the end-product of membrane lipid oxidation. Stability of plasma membrane and maintenance of redox balance is critical to plant drought resistance. Transgenic tomato plants created by *SlHB2-RNAi* had significantly higher expression levels of *CAT1* and *APX1* than NT plants, and showed less membrane damage (Hu et al., 2017). This indicated that *SlHB2* played a negative regulatory role in response to drought and high salt stress. Our findings in this experiment were consistent with those of Hu et al. (2017). However, in plants with high expression of HD-Zip genes such as *ATHB12*-like, *Zmhdz10*, *EsHdzip1*, and *MdHB-7*, the root system was more developed and activity of antioxidant enzymes significantly higher compared to the wild type under drought or salt stress (Zhao et al., 2014, 2020; Zhou et al., 2015a; Wu et al., 2016). This also illustrates the complexity of HD-Zip transcription factors in regulating plant response to drought.

Soybean HD-Zip transcription factor *GmHdz4* was isolated and identified for the first time in this study. The *A*. *rhizogenes* transient transformation system was used to preliminarily verify *GmHdz4* function. The gene negatively regulated drought tolerance in soybean hairy roots by inhibiting lateral root differentiation and weakening osmotic regulation ability and ROS scavenging ability. Our study provides some theoretical basis for further studies on HD-Zip I genes involved in soybean drought tolerance, and indicates the feasibility of breeding new germplasm for drought-tolerant soybean by gene editing. Due to the complex and variable regulation of transcription factors on abiotic stress, the signaling pathway of *GmHdz4* involved in drought response and the specific regulatory mechanism related to root development need to be studied in the future.

## Data availability statement

The datasets presented in this study can be found in online repositories. The names of the repository/repositories and accession number(s) can be found in the article/Supplementary material.

## Author contributions

XZ and GT proposed cloning of and investigating *GmHdz4* gene function under drought conditions. XZ cloned this gene, implemented the molecular and hydroponic work, analyzed the data, and wrote the manuscript. GT, FI, and WZ contributed substantially to the manuscript writing and revisions. WH, YS, JL, and LL participated in the phenotyping identification and root scanning. XC provided plant materials and contributed to paper revisions. All authors contributed to the article and approved the submitted version.

## Funding

This work was supported by the Key Research Foundation of Science and Technology Department of Zhejiang Province (2021C02064-5-5), Hainan Provincial Joint Project of Sanya Yazhou Bay Science and Technology City (320LH033), Key R&D Projects of Zhejiang Province (2021C02057), and Collaborative Innovation Center for Modern Crop Production co-sponsored by Province and Ministry (CIC-MCP).

## Conflict of interest

The authors declare that the research was conducted in the absence of any commercial or financial relationships that could be construed as a potential conflict of interest.

## Publisher’s note

All claims expressed in this article are solely those of the authors and do not necessarily represent those of their affiliated organizations, or those of the publisher, the editors and the reviewers. Any product that may be evaluated in this article, or claim that may be made by its manufacturer, is not guaranteed or endorsed by the publisher.

## Footnotes

^1^ http://www.phytozome.net/index.php/

^2^ http://www.ncbi.nlm.nih.gov/

^3^ http://www.arabidopsis.org/

^4^ http://cbi.hzau.edu.cn/crispr/

## Supplementary material

SUPPLEMENTARY FIGURE S1 PCR amplification of *GmHdz4* M: DNA DL2000 marker; −, negative control; 1 and 2 represent amplification products using soybean total DNA and pUCI-GmHd4 as templates, respectively.

SUPPLEMENTARY FIGURE S2 Overexpression and targeted modification of the *GmHdz4* gene in the soybean hairy roots. **(A)** Schematic diagram of the pTF102–*GmHdz4* vector. The *GmHdz4* gene and *bar* gene were driven by the CaMV 35S promoter. **(B)** The *GmHdz4*-oe hairy root lines were identified by PCR amplification of the *GmHdz4* CDS. The length of the PCR product was 648 bp. **(C)** Schematic illustration of the sgRNA target sequence in the *GmHdz4* gene. The black rectangles represent exons, the black line represents the introns, and the red vertical bars represent the locations of the target sequence. **(D)** Schematic diagram of the pBGK041–*GmHdz4* vector. The Cas9 expression cassette was driven by the CaMV 35S promoter, and the sgRNA cassette was driven by the GmU6 promoter. **(E)** The *gmhdz4* hairy root lines were identified by PCR amplification of *Cas9* gene. The length of the PCR product was 502 bp. **(F)** Mutations induced by sgRNA in *GmHdz4*. The green letter represents the nucleotide insertion and the green dashes represent the deletions, and the labels on the left (*gmhdz4* L1–R1 ~ *gmhdz4* L3-R8) corresponds to each individual hairy root in panel **E**. M represents the DNA marker DL2000. Each individual hairy root on the chimera *GmHdz4-oe* Line 1 ~ 3 and *gmhdz4* Line 1 ~ 3 were examined. -, non-transgenic plant; +, positive control. **(G)** Relative expression of *GmHdz4* among transformed *GmHdz4-oe* chimeric lines, *gmhdz4* chimeric lines, and NT hairy roots. Significant differences are indicated by ^*^ and ^**^ for *p* < 0.05 and *p* < 0.01, respectively, according to Duncan’s test.
