# Supplementary figures and images for: CRISPR/Cas9 mediated gene-editing of GmHdz4 transcription factor enhances drought tolerance in soybean (Glycine max [L.] Merr.)
Source: Front Plant Sci. 2022 Aug 19;13:988505. doi: 10.3389/fpls.2022.988505 (PMC9437544; doi:10.3389/fpls.2022.988505)

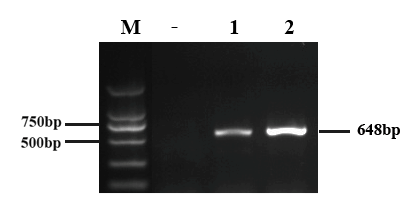

Supplement: SUPPLEMENTARY FIGURE S1 — PCR amplification of GmHdz4 M: DNA DL2000 marker; −, negative control; 1 and 2 represent amplification products using soybean total DNA and pUCI-GmHd4 as templates, respectively. [file Image_1.TIF]

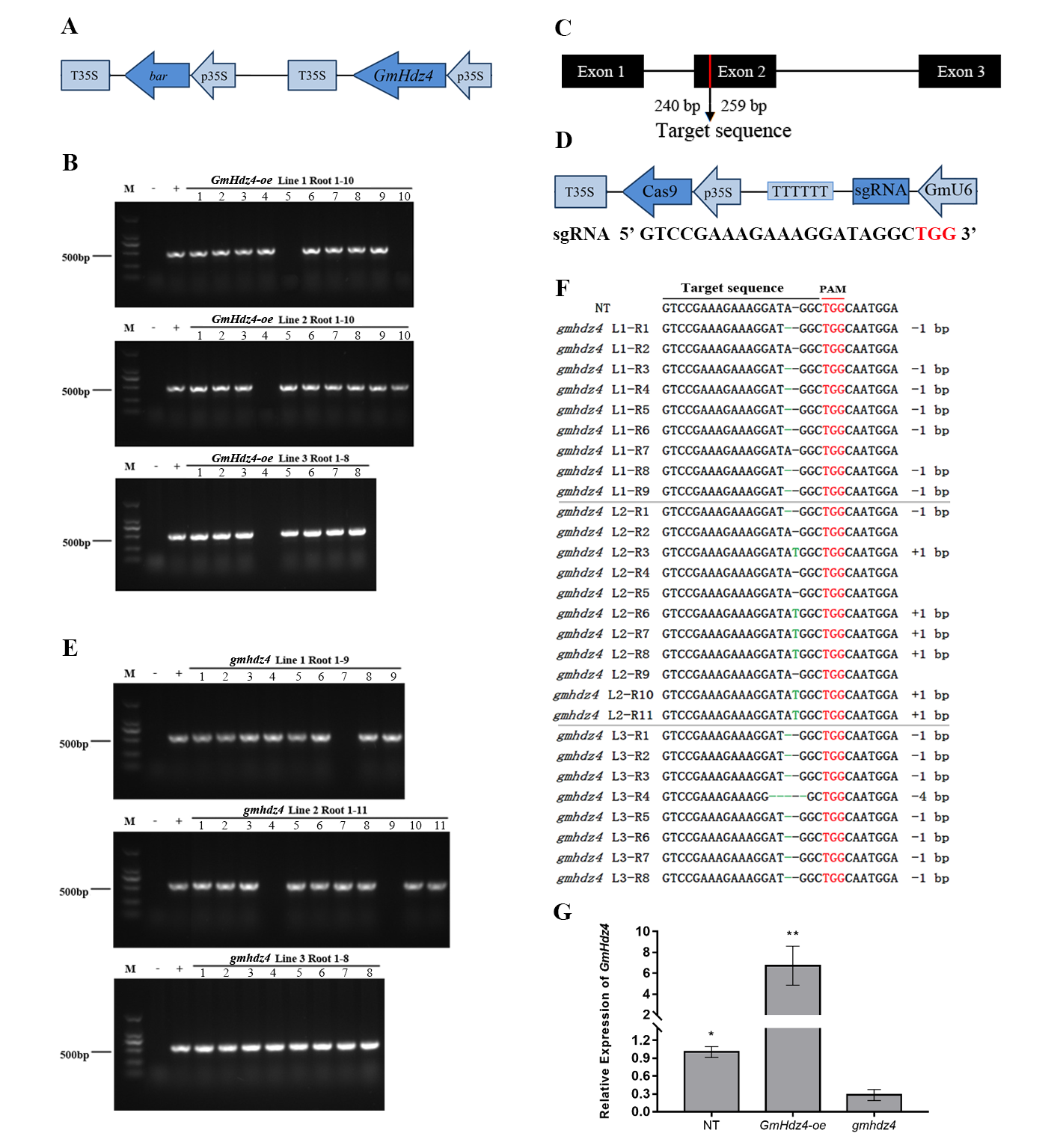

Supplement: SUPPLEMENTARY FIGURE S2 — Overexpression and targeted modification of the GmHdz4 gene in the soybean hairy roots. (A) Schematic diagram of the pTF102–GmHdz4 vector. The GmHdz4 gene and bar gene were driven by the CaMV 35S promoter. (B) The GmHdz4-oe hairy root lines were identified by PCR amplification of the GmHdz4 CDS. The length of the PCR product was 648 bp. (C) Schematic illustration of the sgRNA target sequence in the GmHdz4 gene. The black rectangles represent exons, the black line represents the introns, and the red vertical bars represent the locations of the target sequence. (D) Schematic diagram of the pBGK041–GmHdz4 vector. The Cas9 expression cassette was driven by the CaMV 35S promoter, and the sgRNA cassette was driven by the GmU6 promoter. (E) The gmhdz4 hairy root lines were identified by PCR amplification of Cas9 gene. The length of the PCR product was 502 bp. (F) Mutations induced by sgRNA in GmHdz4. The green letter represents the nucleotide insertion and the green dashes represent the deletions, and the labels on the left (gmhdz4 L1–R1 ~ gmhdz4 L3-R8) corresponds to each individual hairy root in panel E. M represents the DNA marker DL2000. Each individual hairy root on the chimera GmHdz4-oe Line 1 ~ 3 and gmhdz4 Line 1 ~ 3 were examined. -, non-transgenic plant; +, positive control. (G) Relative expression of GmHdz4 among transformed GmHdz4-oe chimeric lines, gmhdz4 chimeric lines, and NT hairy roots. Significant differences are indicated by * and ** for p < 0.05 and p < 0.01, respectively, according to Duncan’s test. [file Image_2.TIF]
