# Supplementary material for: CRISPR/Cas9 mediated gene-editing of GmHdz4 transcription factor enhances drought tolerance in soybean (Glycine max [L.] Merr.)
Source: Front Plant Sci. 2022 Aug 19;13:988505. doi: 10.3389/fpls.2022.988505 (PMC9437544; doi:10.3389/fpls.2022.988505)

The following figures are the results of one-way ANOVA and Duncan's multiple range tests on data of roots structure (RL, Total root length; SA, Superficial area; AD, Average diameter; TN, Tips number. Factors 1, 2, 3 represent NT, mutants, and Oe, respectively).


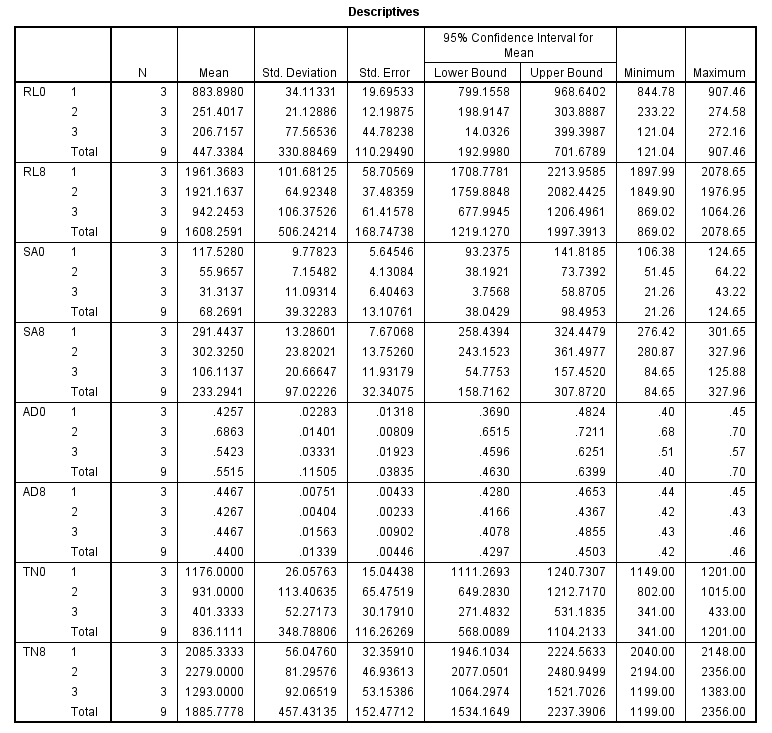

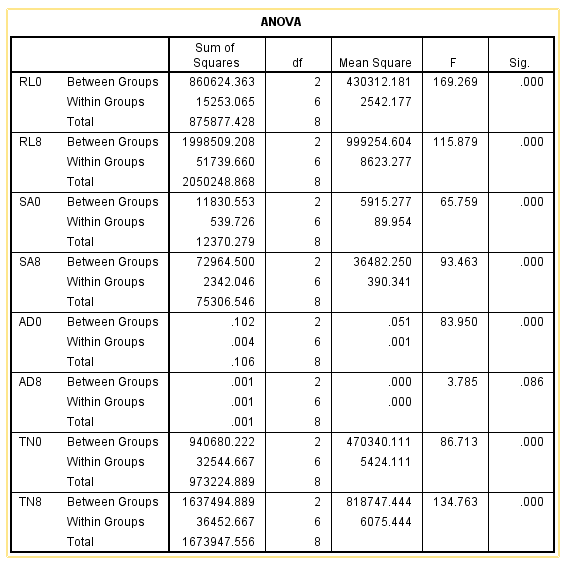

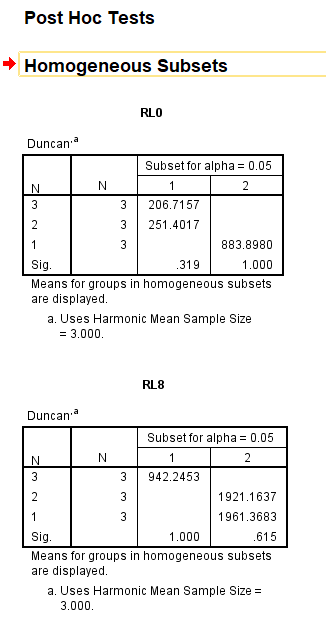

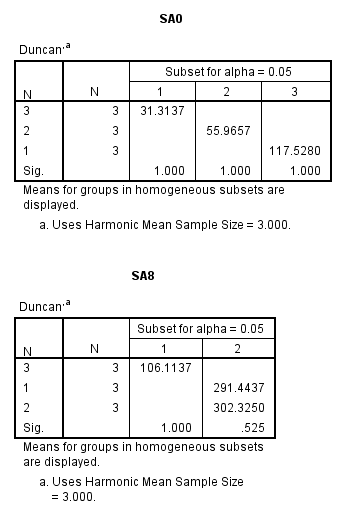

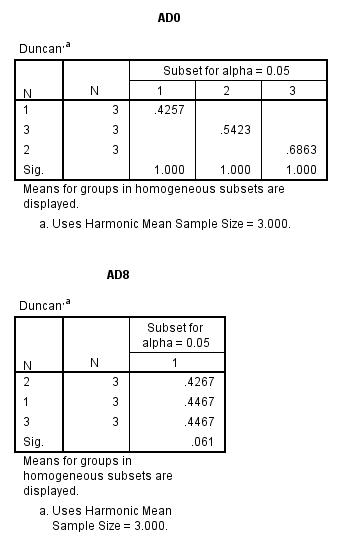

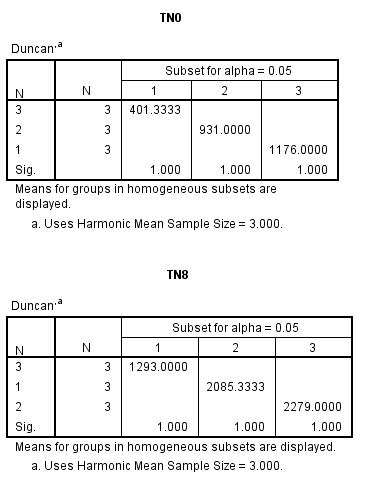

Supplement: Supplementary file 3 [file Table_1.DOCX]
